# Substrate Activation Efficiency in Active Sites of Hydrolases Determined by QM/MM Molecular Dynamics and Neural Networks

**DOI:** 10.3390/ijms26115097

**Published:** 2025-05-26

**Authors:** Igor V. Polyakov, Yulia I. Meteleshko, Tatiana I. Mulashkina, Mikhail I. Varentsov, Mikhail A. Krinitskiy, Maria G. Khrenova

**Affiliations:** 1 Chemistry Department, Lomonosov Moscow State University, 119991 Moscow, Russia; 2 Faculty of Geography, Lomonosov Moscow State University, 119991 Moscow, Russia; 3 Research Computing Center, Lomonosov Moscow State University, 119991 Moscow, Russia

**Keywords:** neural network, AI, hydrolases, QM/MM MD, substrate activation, Laplacian of electron density

## Abstract

The active sites of enzymes are able to activate substrates and perform chemical reactions that cannot occur in solutions. We focus on the hydrolysis reactions catalyzed by enzymes and initiated by the nucleophilic attack of the substrate’s carbonyl carbon atom. From an electronic structure standpoint, substrate activation can be characterized in terms of the Laplacian of the electron density. This is a simple and easily visible imaging technique that allows one to “visualize” the electrophilic site on the carbonyl carbon atom, which occurs only in the activated species. The efficiency of substrate activation by the enzymes can be quantified from the ratio of reactive and nonreactive states derived from the molecular dynamics trajectories executed with quantum mechanics/molecular mechanics potentials. We propose a neural network that assigns the species to reactive and nonreactive ones using the Laplacian of electron density maps. The neural network is trained on the cysteine protease enzyme-substrate complexes, and successfully validated on the zinc-containing hydrolase, thus showing a wide range of applications using the proposed approach.

## 1. Introduction

**Figure 1:**
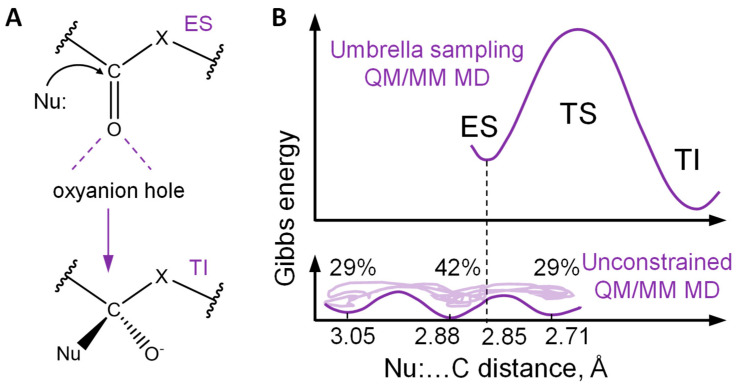
(**A**) The first step of the reaction initiated by the nucleophilic attack of the carbonyl carbon atom of the substrate by the catalytic moiety Nu: X = O,C,N. Dashed violet lines depict hydrogen bonds. ES stands for the enzyme-substrate complex, and TI for the tetrahedral intermediate. (**B**) Gibbs energy profile of the imipenem hydrolysis in the active site of the metallo-β-lactamase NDM−1 (the upper panel) [6]; heterogeneity of the reactant states of the NDM-1-imipenem complex with respect to the distance of the nucleophilic attack [7] (the lower panel).

Substrate activation takes place in the active sites of enzymes. These biocatalytic systems perform chemical reactions that cannot occur in solutions or in the gas phase [1,2]. Among them, numerous reactions are initiated by the nucleophilic attack of the carbonyl carbon atom of a substrate by a catalytic moiety. These reactions take place in hydrolases (EC 3) and result in the cleavage of ester bonds (EC 3.1), peptide bonds (EC 3.4), and other C-N (EC 3.5) or C-C bonds (EC 3.7). These enzymes have two common structural features: an oxyanion hole, and a nucleophilic moiety that initiates reactions (Figure 1A). The oxyanion hole carries amino acid residues that form hydrogen bonds with the oxygen atom of the carbonyl group of the substrate, or a metal cation that forms a coordination bond with the substrate. The oxyanion hole is responsible for lowering the energy barrier of the nucleophilic attack and stabilization of the tetrahedral intermediate with the negatively charged oxygen atom [3,4,5]. Figure 1B clarifies the formulation of the problem as described below using the hydrolysis reaction by the imipenem substrate by the bacterial enzyme metallo-β-lactamase, NDM-1.

There is an urgent need to suggest criteria to discriminate between the reactive and non-reactive species in these enzyme-catalyzed reactions, to avoid brute-force quantum-based calculations of entire Gibbs energy reaction profiles in every application. If a proportion of such calculations can be reduced to evaluate the dynamic features of enzyme-substrate complexes within local areas of a configuration space, then a large reduction of effort would be achieved. This requires the formulation of easily visible criteria based on either geometry parameters, or on electronic structure features. To solve this task, it is required to carry out molecular dynamics calculations with quantum-based potentials, as described, for instance, in Refs [8,9]. However, the use of modern computational approaches based on AI ideas allows one to progress towards facilitating a theoretical analysis. The present work is probably the first approach toward this goal, as described below.

**Figure 2:**
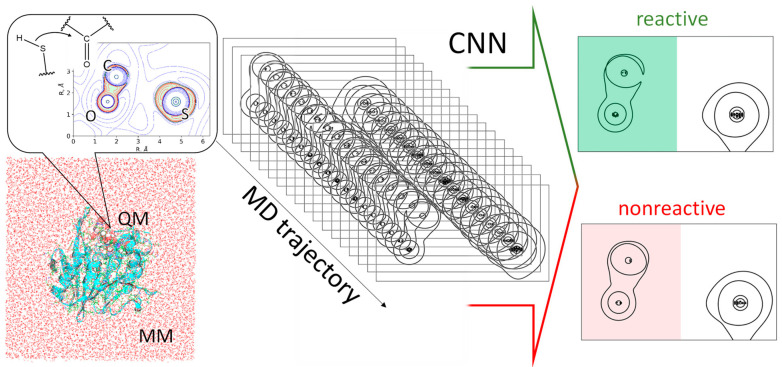
The workflow. QM/MM molecular dynamic trajectories are simulated. Laplacian of electron density maps are calculated in the plane of the carbonyl group and a nucleophilic atom. The inset shows the Laplacian of electron density map with zero (green bold lines), positive (blue dashed lines), and negative (red solid lines) isovalues. Maps are reduced to only zero value contour lines. CNN analyzes only a part of the images comprising a carbonyl group (highlighted green for reactive, and red for non-reactive species) to make a binary classification: reactive or nonreactive.

It has been shown for isolated molecules that the reactivity of unsaturated carbon atoms can be quantified by an electron density analysis [10,11]. Recent theoretical studies have expanded this approach for recognition of substrate activation in the active sites of enzymes [8,9]. The Laplacian of electron density maps demonstrates an electron density depletion area close to the carbonyl carbon atom in the direction of the nucleophilic attack for the reactive (or activated) species (Figure 2). For the non-reactive species, the carbon atom is enveloped by an electron density concentration area. This criterion is already utilized for the binary classification of the species into different hydrolases [8,9]. This electron density feature has been found to be a useful tool for quantifying the substrate activation in related systems [8]. In particular, the substrate specificity of the SARS-CoV-2 main protease (M^pro^) has been explained using this approach [8]. It was shown that substrates with preferable amino acid residues at the P2 position are characterized by a larger proportion of enzyme-substrate (ES) complexes, in which the carbonyl carbon atom of the substrate is in an activated state. To perform this analysis, molecular dynamic (MD) trajectories were calculated with the quantum mechanics/molecular mechanics potentials (QM/MM MD). Then, the maps of the Laplacian of electron density were calculated in the plane of a carbonyl group and a nucleophilic atom at certain MD frames. Geometry-based criteria that allows one to discriminate reactive from non-reactive species were proposed based on the analysis of the structures at these MD frames. Specifically, hydrogen bond distances within the oxyanion hole and the nucleophilic attack distance were identified as practically useful criteria. As a result, a reduction of dimensionality of the entire system to just three geometry parameters was achieved.

Further evidence of the importance of the dynamic features of ES complexes is provided by consideration of the metallo-β-lactamases, NDM-1 and L1, carrying Zn^2+^ cations within the oxyanion hole, and OH^−^ as a nucleophile [7]. The flexibility of the ES complex affects the efficiency of the imipenem substrate activation, and therefore, the energy profile of the subsequent nucleophilic attack. Figure 1B summarizes the related findings of this study for a more flexible system with NDM-1. The Gibbs energy profile of the first reaction step was calculated using QM/MM MD umbrella sampling simulations, with the distance of the nucleophilic attack as a reaction coordinate. Its value at the ES complex equals 2.85 Å. The unconstrained MD simulations in the reactant region demonstrates a broad distribution of the nucleophilic attack distances, which can be described by using at least three normal distributions. One population is characterized by an average value of 2.71 Å, which is smaller than the reaction coordinate at the ES minimum. The most populated proportion (42%) is centered at 2.88 Å, which is the same value as the ES minimum on the Gibbs energy profile. The third proportion is quantified by the mean value of 3.05 Å; this accumulates 29% of the states that correspond to considerably looser ES complexes. In contrast, for a more rigid system with the L1 metallo-β-lactamase, the minimum on the Gibbs energy profile is located at 2.75 Å, and the unconstrained dynamics of the reactant complex demonstrates a narrow distribution of nucleophilic attack distances centered at 2.68 Å. Here, the dimensionality is reduced to only one geometry parameter, which helps to observe differences between two systems with different metallo-β-lactamases. An analysis of the Laplacian of electron density maps also demonstrates notable differences. In the L1-ES, 100% of the considered set of 500 frames shows the activated or reactive carbonyl carbon atom, whereas for the NDM-1-ES this figure is 90%. This is in line with the narrow distribution of distances of the nucleophilic attack in the L1-containing system, and the presence of a proportion of loose conformations in the NDM-1-ES. Also, the importance of activation is pronounced in all of the Gibbs energy profiles of the nucleophilic attack. For tighter L1-containing reactant complexes with a higher degree of activation, the subsequent nucleophilic attack occurs with a lower energy barrier and larger tetrahedral intermediate stabilization, relative to the ES.

Until now, Laplacian electron density maps have been calculated and manually classified for sets of frames extracted from QM/MM MD trajectories, not exceeding 500 frames. In these QM/MM MD simulations, the energies and forces are calculated within the QM subsystem at each trajectory step and then transferred to the MD block. Therefore, the molecular orbitals, and consequently, the electron densities, can also be calculated at each MD step, allowing the Laplacian of electron density analysis to be performed simultaneously at each step. The typical length of the QM/MM MD trajectory is tens of picoseconds, calculated with a 1 fs time step. Utilizing GPUs allows the calculation of around 2 ps per day on a single GPU card, producing 2000 Laplacian electron density maps. It is hard to classify this number of images manually. 

Recent advances in the in silico development of novel proteins, including enzymes with desired functions [12,13,14,15,16,17,18,19], brings further applications to the suggested methodology for estimating enzymatic activity. Once the 3D structure of a novel hydrolytic enzyme is generated, substrates of interest can be inserted into the active site of the molecular model and the activation efficiency can be evaluated.

Herein, we propose an on-the-fly method for estimating reactivity using artificial intelligence algorithms based on image recognition (Figure 2). We propose a convolutional neural network (CNN) that can discriminate reactive from non-reactive species. The model was trained on the enzyme-substrate complexes of the main protease M^pro^ from the SARS-CoV-2 and 6 substrates and then checked on similar systems with three other substrates. For additional evaluation, we utilized a set of the Laplacian electron density maps obtained from two very different hydrolase, each carrying two zinc cations in the active site and a different nucleophile.

## 2. Results and Discussion

**Figure 3:**
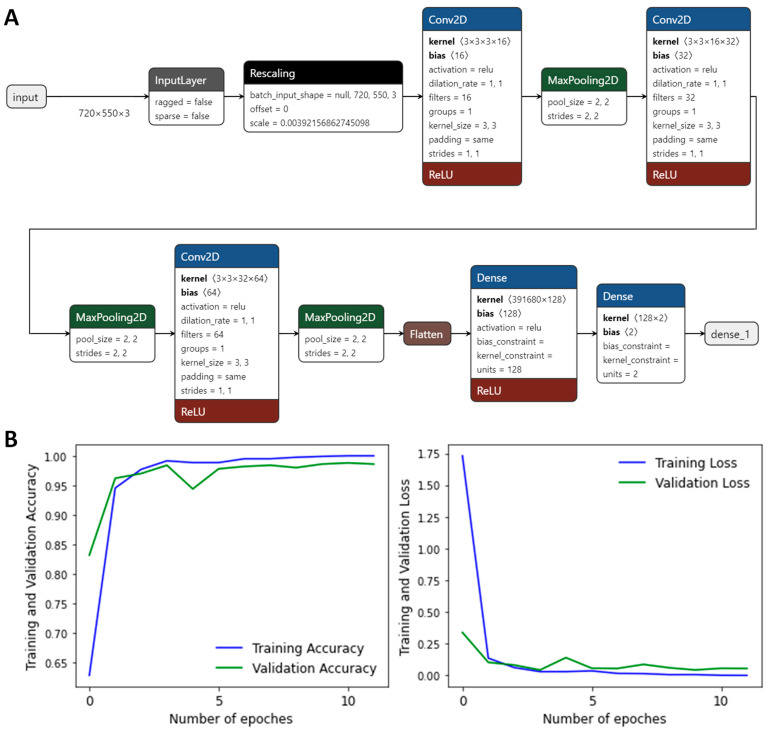
(**A**) The scheme of the CNN for discrimination of reactive and nonreactive states in the active sites of hydrolases. (**B**) The CNN fitting procedure; weights obtained in epoch 10 were utilized.

We obtained datasets of different sizes for training and validation composed of the Laplacian of electron density maps including only the carbonyl groups. All images were initially divided into two groups: reactive and nonreactive. The size of the initial images that carried three atoms was set to 960 × 720. The images were cropped to a size of 550 × 720 and included only the carbonyl group (Figure 2). The datasets for the CNN fitting included 1000, 1500, 2000, 2500, and 3000 images. All the datasets were divided into training and validation subsets in the ratio of 4:1. For all these datasets, the neural network models were built; it was found that a size of 2500 images is optimal for the model fitting. Therefore, the following results only include data obtained using the neural network trained and validated on the dataset of 2500 images. The details of the convolution neural network are presented on Figure 3A; Tensorflow and Keras were utilized within the Python framework. The neural network in Keras format, Python 3.6 scripts, and all the datasets can be found at the ZENODO. To avoid overfitting the CNN, we utilized an early stopping callback function. Our model monitors a validation loss during the fitting procedure and allows recover of optimal weights if the validation loss starts growing from the minimal value. The accuracy of the CNN was found to be higher than 0.98 with respect to the validation set (Figure 3B) and 1.00 for the training set. The next step was application of the CNN to the other model systems.

**Figure 4:**
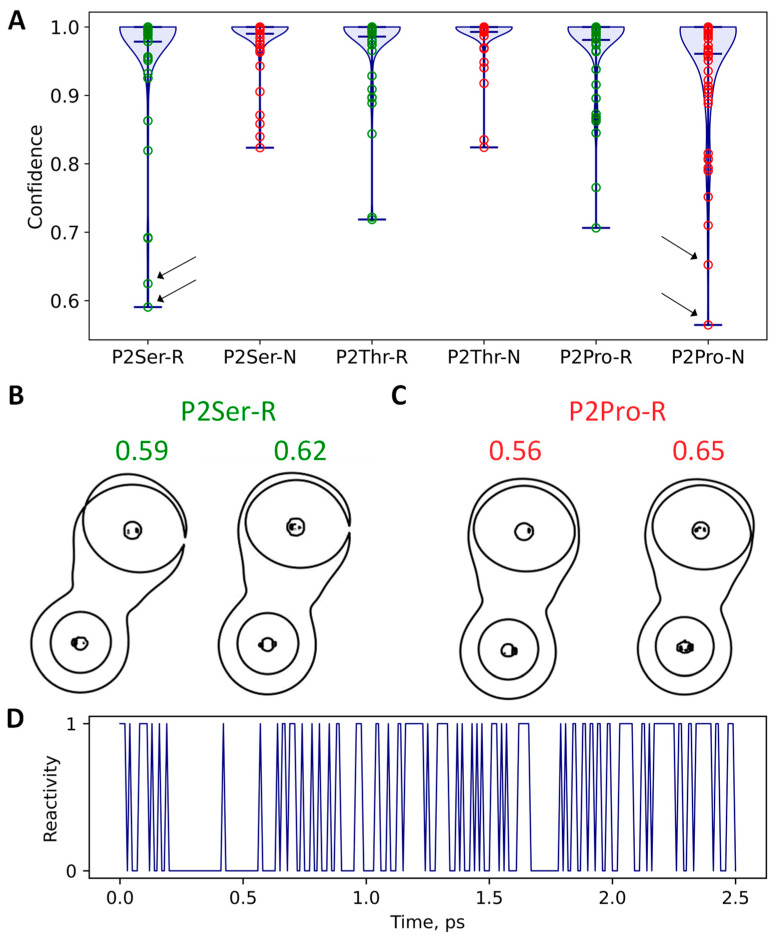
Analysis of images obtained from the frames of QM/MM MD trajectories of enzyme substrate complexes of the M^pro^ and substrates with Pro, Ser, or Thr at P2. (**A**) Confidence of reactivity determination for the reactive (marked with R, green circles) and non-reactive (marked with N, red circles) species. Arrows demonstrate the points corresponding to the images from panels (**B**,**C**) with the lowest values of confidence. (**B**) Images with the lowest confidence of determination among the reactive species. (**C**) Images with the lowest confidence of determination among the non-reactive species. (**D**) Alternation of reactive (with 1 value at y-axis) and non-reactive (with 0 value at y-axis) species along the QM/MM MD trajectory of the reactant complex of the M^pro^ and a substrate containing an Ile residue at P2.

The CNN model was trained using images obtained from the structures from the QM/MM MD simulations of the enzyme-substrate complexes, with the substrates containing one of the following residues at P2: Ala, Phe, Ile, Gln, Tyr, or Trp. We performed additional simulations with other substrates containing Ser, Thr, or Pro at P2 to test the CNN. For each system, we manually selected 100 images corresponding to the reactive species, and 100 images with the nonreactive states. These images were classified using the CNN model. For systems containing Ser or Thr at P2, all the images were classified correctly. For the Pro containing systems, there were both false positive and false negative results. Six reactive species were classified as nonreactive and eight non-reactive species as reactive. Figure 4A demonstrates the violin plots of the confidence of prediction of the reactive/non-reactive species. All average confidence values are larger than 0.96. The two highest values correspond to the datasets with the non-reactive species with either a Ser or Thr residue at P2; these exceeded 0.99. We analyzed the images that corresponded to the lowest confidence of reactivity prediction; these were two reactive ones for the systems with a serine-containing substrate (Figure 4B), and two nonreactive with the system containing a proline residue at P2 (Figure 4C). For the reactive species, it seems that the difficulties for the CNN were due to the small area of electron density depletion. However, the non-reactive species looked similar to the other images of the same type making this result non-evident.

We analyzed whether the transitions between reactive and non-reactive species are periodic processes or stochastic. Figure 4D demonstrates an example of such transitions in a reactant complex of the M^pro^ and a substrate containing an Ile residue at P2, with the proportion of reactive species around 1/2. The sequence of transitions is random with the duration of either of the two states being shorter and longer without any regularity.

**Figure 5:**
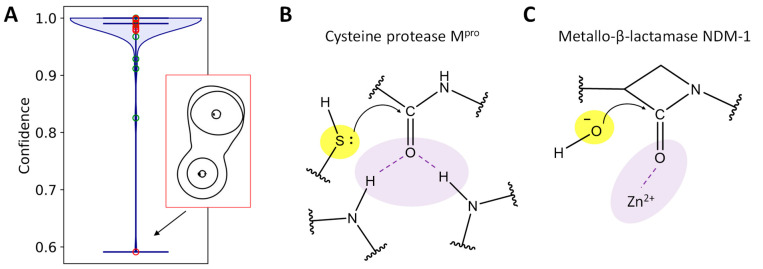
(**A**) Confidence of reactivity determination for the dataset from the NDM-1-imipenem complex containing both reactive (green circles) and non-reactive (red circles) species. A nucleophile (yellow) and an oxyanion hole (lavender) in the cysteine protease M^pro^ (**B**) and metallo-β-lactamase NDM-1 (**C**).

To study transferability of results, we obtained another computational experiment and utilized a set of 100 random frames from the QM/MM MD trajectory of the enzyme-substrate complex of the bacterial enzyme metallo-β-lactamase NDM-1 and imipenem antibiotic. This complex differs in both respects from the M^pro^-oligopeptide complexes (Figure 5B,C). In NDM-1, the nucleophilic moiety is the hydroxide anion. The oxygen atom performs a nucleophilic attack instead of the sulfur atom of the cysteine residue in the M^pro^. The oxyanion hole is formed only by the Zn^2+^ cation instead of two hydrogen bonds, with the main chains of Cys145 and Gly143 in the M^pro^. Among 100 images, only two were classified incorrectly (two non-reactive species were recognized as reactive by the CNN). For those classified correctly (Figure 5A), we obtained a distribution of the confidence of determination. The average confidence value is 0.99 and only two images are determined with a confidence lower than 0.9. The outlier is the image with the confidence of determination being 0.59, and it is depicted in Figure 5A. This can be explained as the electron density concentration area in the region of the nucleophilic attack being narrow, similar to the reactive species.

## 3. Models and Methods

We utilized a model system composed of the SARS-CoV-2 M^pro^ and the chromogenic oligopeptide substrate from ref [8]. The source of coordinates of heavy atoms of the M^pro^ was a crystal structure (PDB ID: 6LU7) [20]. We considered a set of enzyme-substrate complexes studied in ref [8] and those obtained in this study. These included oligopeptides containing Ile, Phe, Ala, Gln, Tyr, Thr, Ser, Pro, and Trp residues at P2 position on the substrate. The sequence of the entire fluorogenic substrate is as follows: ACE–P4 (Val)–P3 (D-Tyr)–P2 (X)–P1 (Gln)-ACC, where ACE means that the N-terminal amino acid residue is acetylated, and ACC is a fluorescent tag 7-amino-4-carbamoylmethylcoumarin. The systems that were not considered in ref [8] were manually prepared by substitution of Ile at P2 on the substrate in the enzyme-substrate complex. Thus, the prepared systems were preliminary equilibrated similarly to ref [8]. During the preliminary steps, classical MD simulations were performed using the NAMD 2.13 [21] program. We utilized the CHARMM36 [22,23] force field parameters for protein atoms, the CGenFF [24] parameters for ACC, and the TIP3P [25] parameters for water molecules. The 1000 steps minimization followed by the 1 ns run was utilized to equilibrate the system. Harmonic potentials were added for two noncovalent interactions along classical MD trajectories. Namely, we constrained a distance between the C atom of the substrate and the S atom of the Cys145 (centered at 2.9 Å with a force constant of 80 kcal∙mol^−1^∙Å^−2^) and a hydrogen bond between the hydrogen atom of the side chain of Cys145, and the side chain nitrogen atom of His41 (centered at 1.9 Å with a force constant of 20 kcal∙mol^−1^∙Å^−2^). These constraints were applied only in the classical MD simulations.

The last frames of the corresponding classical MD trajectories were utilized to create a starting set of coordinates for the MD simulations with the QM/MM potentials. The MM subsystems were described with the same force field parameters as in the preliminary classical MD simulations. The QM subsystems were described at the Kohn-Sham DFT level with the PBE0 [26] functional, with the 6–31 G** basis set and an empirical dispersion correction D3 [27]. It included the fragment of the substrate (a carbonyl fragment of P2 (X) and all the atoms from P1 (Gln) and ACC), the side chain of His41, Cys145 (catalytic dyad) and Gly143, and the CONH groups of the backbone connecting Asn142 with Gly143, and Ser144 with Cys145 (oxyanion hole) giving 73 atoms in total. Hydrogen link atoms were added to the covalent bonds between the QM and MM subsystems. The TeraChem program [28] was used to compute energies and forces in the QM subsystem. The QM/MM MD simulations were performed within the TeraChem-NAMD interface [29] using an electrostatic embedding scheme, i.e., the electron density of the QM subsystem was polarized by point charges of the MM subsystem due to the contributions of the MM atomic charges to the one-electron part of the QM Hamiltonian. The 10 ps MD trajectories were calculated for each enzyme-substrate complex.

Both classical and QM/MM MD calculations were performed with a 1 fs integration time step in the NPT ensemble at *p* = 1 atm and T = 300 K. The cutoff distances were 12 Å for both the electrostatic and van der Waals interactions, with switching to the smoothing function at 10 Å. The VMD software [30] was used for preparation of the model systems and subsequent analysis of the molecular dynamics (MD) trajectories.

Also, a set of 100 MD frames were extracted from the MD trajectory of the NDM-1 complex with the imipenem from ref [6]. The details of the calculations can be found in the original paper [6]. The QM/MM protocol was the same as for the systems with the M^pro^, as discussed above.

The Laplacian of the electron density, ∇^2^*ρ*(**r**), [8,31,32,33,34] was calculated at certain MD frames in the plane containing the carbonyl group of the substrate, and an atom of the nucleophilic species that can attack a carbonyl carbon (a sulfur atom of the Cys145 in the M^pro^ or an oxygen atom of the hydroxide anion in the NDM-1). In these maps (Figure 2), we can observe electron density concentration regions with ∇^2^*ρ*(**r**) < 0 (electrophilic sites) and electronic density depletion areas with ∇^2^*ρ*(**r**) > 0 (nucleophilic sites). An electron density analysis was performed in the Multiwfn program package [35]. Usually, a set of contour lines with different isovalues are put to the Laplacian of electron density contour maps (Figure 2). In our case we were only interested in the contour line ∇^2^*ρ*(**r**) = 0 and its behavior in the carbonyl carbon atom region. Therefore, we drew a minimalistic plot with the only contour line with the Laplacian of electron density equal to zero and cropped the image remaining with only a carbonyl group in each image (color-filled parts of images on Figure 2). We were not interested in the structure of the nucleophile; moreover, both S and O atom can act as nucleophiles and the differences in their electronic structures can be recognized by the CNN incorrectly. The sample input files for the Laplacian of electron density calculations using the Multiwfn and Python scripts for visualization can be found at the ZENODO. The suggested way of data analysis is transferable and can be applied to any model system of hydrolytic enzymes that initiate reactions with the nucleophilic attack of the carbon atom. The images of ∇^2^*ρ*(**r**) maps obtained by the Multiwfn are similar for different systems; therefore, the designed algorithm can be easily applied for systems of such type.

## 4. Conclusions

We obtained a convolutional neural network that can automatically recognize whether a substrate is reactive (or activated) in the active site of a hydrolase, or not. This model was trained on the enzyme-substrate complexes of the M^pro^ with different substrates and examined using similar models with other substrates of this enzyme. Another dataset was prepared from the molecular dynamic trajectory of the enzyme substrate complex of the metallo-β-lactamase, NDM-1, and imipenem (an antibiotic). The neural network recognized both reactive and non-reactive species in all the datasets, with a low percentage of errors, that is the substrates under consideration can be, at least, semi quantitatively divided into reactive, non-reactive and intermediate types. This technique can be utilized for the on-the-fly determination of substrate activation in the active site of hydrolytic enzymes of different types, along with the molecular dynamic trajectories calculated with the combined quantum mechanics/molecular mechanics potentials. The growing interest in the suggested methodology is due to the development of AI-based methods to design novel enzymes. It can be utilized to perform quick scan of substrates and to determine their activation efficiency using the developed enzyme.

## Data Availability

The original contributions presented in the study are included in the article, further inquiries can be directed to the corresponding author. Collection of scripts, datasets and CNN in Keras format are available in the Zenodo and can be accessed via https://doi.org/10.5281/zenodo.6964866.
